# FIR-preconditioning promotes Akt-mTOR-exosome manufacture in cooperation with MITF to boost resilience of rat bone marrow-derived stem cells

**DOI:** 10.1016/j.heliyon.2023.e15003

**Published:** 2023-04-07

**Authors:** Yun-Mi Jeong, Weon Kim

**Affiliations:** a Department of Mechanical Engineering, Tech University of Korea, 237 Sangidaehak Street, Si-heung City, Republic of Korea; b Division of Cardiology, Department of Internal Medicine, Kyung Hee University Hospital, Kyung Hee University, Seoul, Republic of Korea

**Keywords:** Far-infrared irradiation, Bone marrow-derived mesenchymal/stromal cells, MITF, Akt/mTOR/exosome manufacture, Cellular resilience

## Abstract

A previous study from our laboratory observed the protective effects of far-infrared irradiation (FIR) on bone marrow-derived stem cells (BMSCs) against oxidative stress. However, it remains unknown precisely how FIR influences BMSC survival. We identify an unexpected route among the expression of MITF, BCL2, mTOR, and exosome in FIR-preconditioned BMSCs. MITF siRNA demonstrated that loss of MITF expression not only inhibited cell proliferation but also reduced the FIR-mediated expression of mTOR, BCL2, and exosome. mTOR signaling pathways have been implicated in cell growth, proliferation, and survival. We also found that rapamycin, a potent and selective inhibitor of mTOR, when combined with MITF siRNA, repressed FIR-mediated CD63 and BCL2 expression. In addition, FIR-preconditioned BMSCs demonstrated more tolerance in multiple stressful environments than untreated BMSCs. The elevated exosomes in conditioned medium derived from FIR-preconditioned BMSCs also repaired H9c2 cells that sustained cellular damage after subjected to an array of environmental stress conditions. Taken together, these results reveal a possible mechanism about how FIR-preconditioned BMSCs and its conditioned media could contribute to cellular resilience during environmental changes via MITF-Akt-mTOR associated with exosome manufacture. FIR preconditioning could thus complement and improve therapeutic applications of BMSCs on outcomes of various disorders.

## 1 Introduction

The Microphthalmia family of bHLH-LZ transcription factors (MiT/TFE) consists of four members: microphthalmia-associated transcription factor (MITF), TFEB, TFE3, and TFEC. They play central roles in the regulation of cellular processes including lysosomal homeostasis and autophagy induction [1]. In particular, MITF bind to M-boxes (5′-TCATGTG-3′) and symmetrical DNA sequences (E-boxes, 5′-CACGTG-3′), and then upregulates or downregulates the expression of targeted genes with central roles in cell differentiation, invasion, senescence, metabolism, proliferation, survival, and DNA damage repair [1]. Although the specific roles of MITF in bone mesenchymal/stromal stem cells (BMSCs) have not yet been identified, there is evidence that suggests MITF might serve as a multifunctional modulator in exosome generation and regulating autophagy in several cell lines [[2], [3], [4], 24]. For example, forskolin, a known a-melanocyte-stimulating hormone agonist, increases exosome production in MSCs, including upregulation of MITF [2]. MITF binds the CLEAR-box element in the promoters of lysosomal and autophagosomal genes in melanoma cells and melanocytes [3]. Additionally, MITF is involved in chemoresistance to cisplatin in A549 lung cancer cells through regulating lysosomal biogenesis and autophagy (Li W et al., 2021).

BMSCs are easily obtained from the small aspirates of bone marrow [5]. BMSCs have been reported to be utilized in regenerative medicine research due to their differentiation potential or the paracrine factors of their extracellular vesicles including exosomes [6]. A recent paper has demonstrated that exosomes from conditioned media derived from BMSCs increase bone regeneration by promoting angiogenesis [7]. Another paper reports that human BMSC-derived exosomes effectively promote cutaneous wound healing via TGF-b/Smad signaling pathways [8]. Despite the beneficial effects of BMSCs *in vitro* and *in vivo*, several limitations still impede their use in clinical trials. A previous study from our laboratory has reported that preconditioning with far-infrared (FIR) radiation promotes proliferation, cell survival, and migration of rat bone marrow-derived stem cells through CXCR4-ERK pathways [9]. Of note, when BMSCs are preconditioned by FIR prior to being subjected to oxidative stress, the cell survival ratio of the FIR-preconditioned BMSCs is higher than that of the control [9]. However, the mechanism underlying how BMSCs preconditioned by FIR are able overcome oxidative stress remains unclear. The present study first explores how FIR preconditioning results in this cellular resilience in BMSCs. Then we investigate whether and to what extent FIR-preconditioned BMSCs can cope under different environmental conditions. Finally, we validate whether conditioned medium (CM)-derived FIR-preconditioned BMSCs can augment the resilience of H9c2 cells subjected to extreme environmental conditions.

## 2 Results

### 2.1 FIR preconditioning offers the opportunity to amplify cell survival signaling pathways and MITF expression in BMSCs

**Fig. 1:**
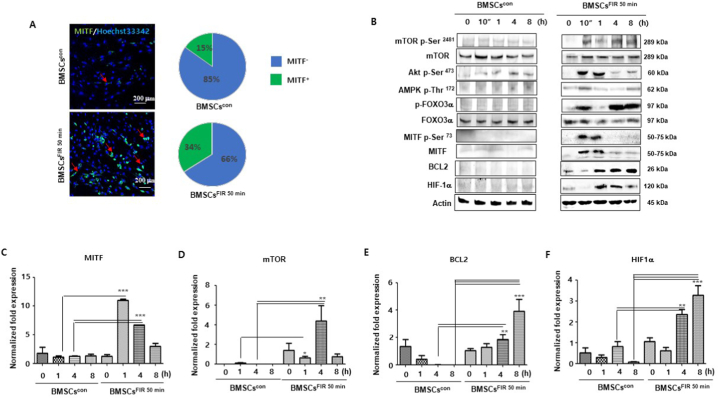
**FIR preconditioning affects the activation of MITF and Akt-mTOR signaling pathways at protein and mRNA levels.** (A) After FIR 50 min preconditioning, these cells were incubated in starvation media for 1 h and then fixed. IFS were analysis showing the expression of MITF (green) in BMSCs^con^ and BMSCs^FIR 50 min^ Hoechst33342 used for nuclear staining. Scale bars, 200 μm. Diagram shows the number of MITF^−^ (blue) and MITF^+^ (green) cells in BMSCs^con^ and BMSCs^FIR 50 min^ (Red arrow to MITF^+^ cells). (B) Western blot analysis was performed with antibodies to detect FIR-mediated cellular signaling pathways in each group at the indicated time points. Actin is used as a loading control. (C - F) qRT-PCR bar graphs to quantify the expression of the targeted genes in BMSCs^con^ and BMSCs^FIR 50 min^. All data represent the mean ± SD of triplicated assays expressed as percentages of the BMSCs^con^. *P<0.05, **P<0.01, ***P<0.01 versus corresponding BMSCs^con^ in the indicated time point using Student's *t*-test. (For interpretation of the references to colour in this figure legend, the reader is referred to the Web version of this article.)

Representative confocal images and diagram graphs showed enrichment of the MITF ^+^ population in BMSC ^FIR 50 min^, indicating a high level of MITF expression (Fig. 1A). Finding that FIR preconditioning led to the expression of MITF was intriguing because the role of MITF in BMSCs remains unclear. To determine whether FIR preconditioning affects the expression of MITF and its related cellular responses in BMSCs, we focused on one set of Akt/mTOR-related signaling pathways, which have been recognized as a key regulator of survival signaling pathways [1,10]. As shown in Fig. 1A, phosphorylation of Akt Ser^473^ was markedly activated 10 min after FIR 50 min preconditioning and peaked at 1 h (Fig. 1B). Increased phosphorylation of mTOR Ser^2481^, AMPK Thr^172^, and the Akt downstream target FoxO1/3 A Thr^24/32^ was also detected after 10 min, whereas the phosphorylation of mTOR Ser^2481^, AMPK Thr^172^, and FoxO1/3A Thr^24/32^ decreased after 1h, then recovered after 4 h (mTOR Ser^2481^ and FoxO1/3A Thr^24/32^) or 8 h (AMPK Thr^172^) (Fig. 1B). FIR 50 min preconditioning increased the expression of BCL2 and HIF-1α after 1 h (Fig. 1B). While exploring regulation of cellular Akt/mTOR signaling networks in the FIR-preconditioned BMSCs, we also observed that the phosphorylation of MITF S^73^ and the expression level of MITF dramatically increased 10 min to 1 h after FIR preconditioning (Fig. 1B). qRT-PCR assay further confirmed gene expression of mTOR, MITF, BCL2, and HIF-1α (Fig. 1C–F and Fig. S1). These results indicate that FIR-preconditioning is associated with Akt/mTOR-related signaling pathways and the expression of MITF-BCL2-HIF-1α required for cell survival.

### 2.2 FIR preconditioning promotes exosome production

**Fig. 2:**
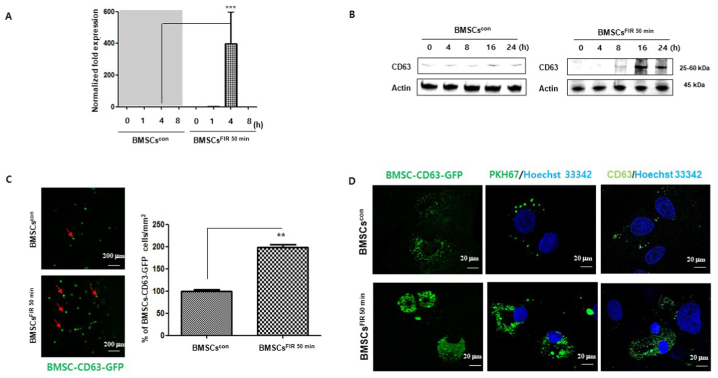
**Exosome product in BMSCs is elevated by FIR preconditioning.** (A) After FIR ^50 min^ preconditioning, total RNA was prepared at the indicated time point. qRT-PCR bar graphs to quantify the expression of the CD63 in BMSCs^con^ and BMSCs^FIR 50 min^. RPL32 was used as control. All data represent the mean ± SD of triplicated assays expressed as percentages of the BMSCs^con^. Data was analyzed using one-way analysis of variance (ANOVA) followed by Student's *t*-test. ***P<0.001 versus corresponding controls (gray box). (B) Western blot analysis of CD63 in the indicated time points. Actin was used as a loading control. (C) BMSCs were exposed with or without FIR 50 min at 3 days post-transfection of pCT-CD63-GFP. Confocal microscope was used to detect the expression of CD63 (green) (Red arrow to CD63-expressed cells). Scale bars, 200 μm. Graph depicting the percentages of CD63^+^ cells in BMSCs^con^ and BMSCs^FIR50 min^. All data represent the mean ± SD of triplicated assays expressed as percentages of the BMSCs^con^. **P<0.01 versus corresponding control using Student's *t*-test. (D) Localization of CD63 (green) in BMSCs^con^ and BMSCs^FIR50 min^ using pCT-CD63-GFP Cyto-Tracer transfection, live cell imaging of PKH67, and IFS with anti-CD63 antibodies. Hoechst33342 nuclei (blue) are visualized. Scale bars, 20 μm. (For interpretation of the references to colour in this figure legend, the reader is referred to the Web version of this article.)

The protective function of the Akt/mTOR-signaling network has been known to coordinate fundamental cellular processes including synergies in exosomes and autophagy pathways [10,11]. BMSCs ^FIR 50 min^ exhibited a high expression of exosome marker CD63 in comparison with BMSCs ^con^ at both mRNA and protein levels (Fig. 2A and B, and Fig. S2). To further confirm exosome production in BMSCs ^con^ and BMSCs ^FIR 50 min^, we generated CD63-GFP-expressing BMSCs using a pCT-CD63-GFP vector. As expected, the level of CD63-GFP expression in BMSCs ^FIR 50 min^ was twofold greater than in BMSCs ^con^ (Fig. 2C). Using PKH26, IF staining with anti-CD63 antibodies, and CD63-GFP-expressing BMSCs, these observations further confirmed that production of exosome in BMSCs was more elevated under FIR preconditioning versus the control (Fig. 2D). These results indicate that boosting the exosome production derived from FIR-preconditioned BMSCs may be intertwined with both Akt/mTOR signal pathways and MITF-driven prosurvival signals.

### 2.3 MITF is a candidate molecular switch between mTOR-linked exosome manufacture and to cellular resilience of FIR-preconditioned BMSCs

**Fig. 3:**
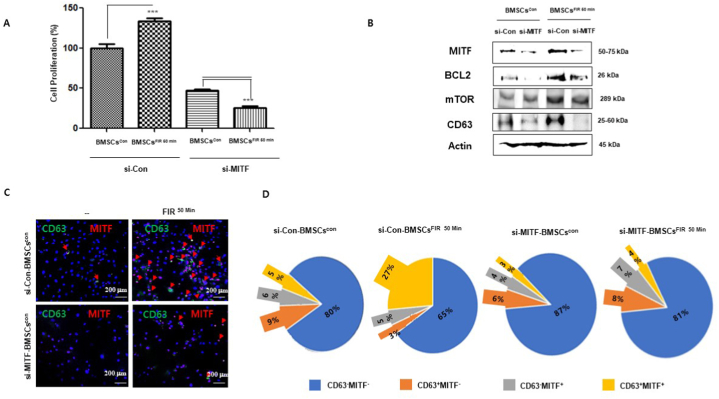
**Inactivation of MITF inhibits cell proliferation of FIR-preconditioned BMSCs via BCL2, mTOR and exosomes.** (A) Bar graphs to calculate the cell proliferation of the indicated group in BMSCs^con^ and BMSC^FIR 50 min^, in the absence or presence of si-MITF. The cell proliferation was determined by the crystal violet staining methods. All data represent the mean ± SD of triplicated assays expressed as percentages of the BMSCs^con^. Data was analyzed using Student's *t*-test. ***P<0.001 versus each corresponding controls. (B) After loss of MITF expression, Western blot analysis of MITF, BCL2, mTOR, and CD63 expression in each group. Actin was used as a loading control. (C) Confocal fluorescence images of CD63 and MITF in each group. CD63 (green), MITF (red), and Hoechst33342 nuclei (blue) are visualized (Red arrows to CD63^+^/MITF^+^ cells). Scale bars, 20 μm. (D) Diagram indicating the percentages of CD63^−^MITF^-^(․), CD63^+^MITF^−^ (․), CD63^−^MITF^+^(․), and CD63^+^MITF^+^ (․) in si-Con BMSCs^con^, si-Con BMSCs^FIR 50 Min^, si-MITF BMSCs^con^, and si-MITF BMSCs^50 Min^. (For interpretation of the references to colour in this figure legend, the reader is referred to the Web version of this article.)

Although details regarding the timing and mechanisms of MITF activity during cellular resilience in BMSCs are not entirely understood, the interaction between MITF and exosomes manufacture in cells has been found to influence cellular homeostasis including cell proliferation and survival [1]. If MITF activity switches on exosome-mTOR-mediated cell survival signaling in BMSCs by FIR preconditioning, then MITF would be driving cellular responses that contribute to the survival and proliferation of BMSCs. To test this, BMSCs were transfected with a scrambled si-Con or si-MITF before FIR preconditioning. qRT-PCR and Western blot analysis demonstrated that transfection with si-MITF led to loss of MITF expression compared with a scrambled si-Con (Fig. 3A and Fig. S3A). After transfection at 24 h, no significant difference was observed in the cell viability between si-Con- and si-MITF-transfected BMSCs (Fig. S3B). BCL2 protein and CD63 decreased in MITF-depleted BMSCs (Fig. 3A). High expression of BCL2 and CD63 in BMSCs ^FIR 10 min^ was reduced in MITF siRNA BMSCs in comparison to si-Con BMSCs. The expression of mTOR was not affected by si-MITF. si-MITF also prevented mTOR changes in BMSCs ^FIR 10 min^ (Fig. 3A). We turned to cell proliferation using an EZ-Cytox cell viability assay kit for detecting the relationship between loss of MITF and proliferation of BMSCs in the presence and/or absence of FIR preconditioning. si-Con BMSCs ^FIR 50 min^ exhibited an approximately 1.5-fold increase in cell proliferation, compared with si-Con BMSCs ^con^ (Fig. 3B). Loss of MITF significantly decreased cell proliferation in both BMSCs ^con^ and BMSCs ^FIR 50 min^ (Fig. 3B). Cell proliferation was lower for si-MITF BMSCs ^FIR 50 min^ than for si-MITF BMSCs ^con^.

**Fig. 4:**
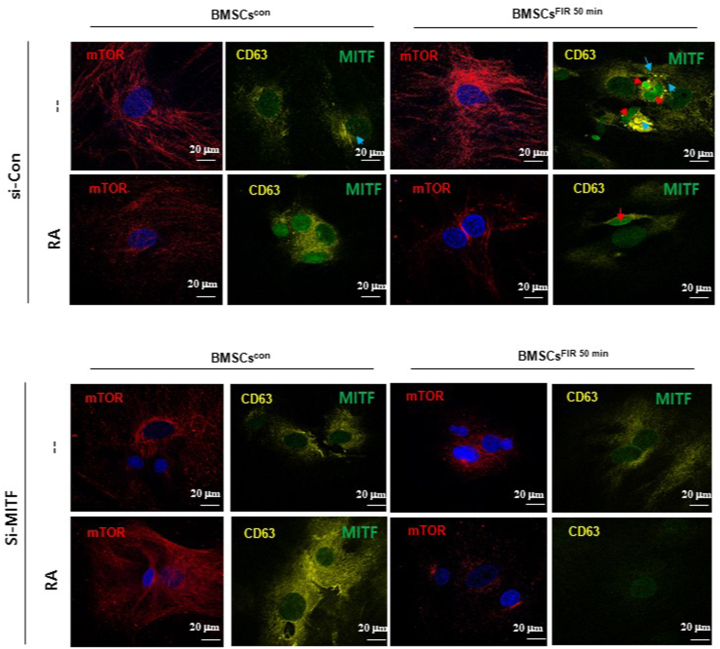
**Regulation of mTOR, MITF, and exosome by FIR with or without si-MITF and rapamycine.** Confocal fluorescence images of mTOR, CD63, and MITF in si-Con BMSCs^con^, si-Con BMSCs^FIR 50 Min^, si-MITF BMSCs^con^, and si-MITF BMSCs^50 Min^ with or without RA (rapamycine). MITF (green), mTOR (Red), CD63 (yellow), and Hoechst33342 nuclei (blue) are visualized. Scale bars, 20 μm. (For interpretation of the references to colour in this figure legend, the reader is referred to the Web version of this article.)

To evaluate cellular changes upon exosome manufacture in si-Con BMSCs and si-MITF BMSCs via FIR preconditioning, exosome and MITF expression were detected using IFS with anti-CD63 and MITF antibodies. Representative confocal images showed more enrichment of CD63^+^ MITF^+^ in si-Con BMSCs ^FIR 50 min^ than in CD63^+^ MITF^+^ in si-MITF BMSCs ^FIR 50 min^ (Fig. 3C and D). These results indicate that the interplay between MITF and mTOR-exosome manufacture has a significant role in improving the cell proliferation of FIR-preconditioned BMSCs. We next used rapamycin to further verify whether there is a relationship between MITF and the mTOR-exosome manufacturing process in FIR preconditioned BMSCs. We found that rapamycin abrogated the expression of mTOR, MITF and exosome in the BMSCs ^FIR 50 min^ (Fig. 4 and Fig. S4). Furthermore, both rapamycin and loss of MITF have clear negative effects on mTOR and exosome expression in the BMSCs ^FIR 50 min^ (Fig. 4). These findings indicate that MITF might be involved in BMSC proliferation via akt-mTOR-exosome manufacturing.

### 2.4 FIR-preconditioned BMSCs and their conditioned medium improve BMSC and H9c2 ability to adapt and survive under extreme environmental conditions

**Fig. 5:**
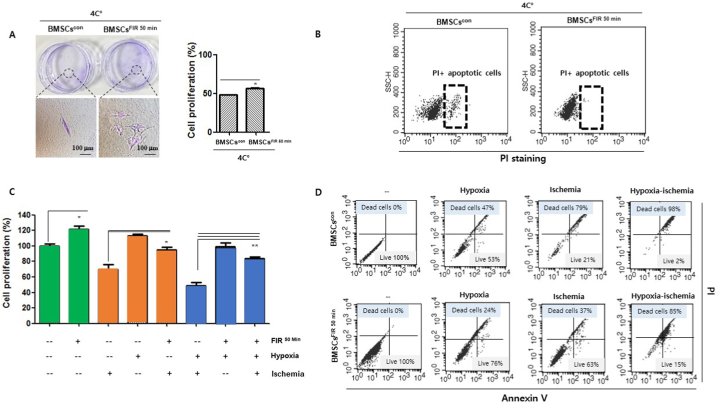
**The resilience of proliferation capacity of FIR-preconditioned BMSCs after extreme environmental conditions.** (A) Image of crystal violet-stained culture dish and bar graph of the survival rate of BMSCs^con^, and BMSCs^FIR 50 Min^ after 4 °C. All data represent the mean ± SD of triplicated assays expressed as percentages of the BMSCs^con^. Data was analyzed using Student's *t*-test. *P<0.05 versus each corresponding controls. (B) The apoptotic cells in BMSCs^con^, and BMSCs^FIR 50 Min^ after 4 °C were analyzed by flow cytometry using a PI staining in the live cells, as described in the Materials and Methods. (C) Graph of the recovery of proliferation of BMSCs^con^, and BMSCs^FIR 50 Min^ after extreme environmental conditions. Data was analyzed using one-way analysis of variance (ANOVA) followed by Tukey's *post hoc tests*. **P<0.01 and *P<0.05 versus corresponding controls (green, orange, blue box). (D) PI/annexin V assays to detect the apoptotic cells in BMSCs^con^ and BMSCs^FIR 50 Min^, after extreme environmental conditions (blue box, apoptotic/dead cells; gray box, live cells). (For interpretation of the references to colour in this figure legend, the reader is referred to the Web version of this article.)

**Fig. 6:**
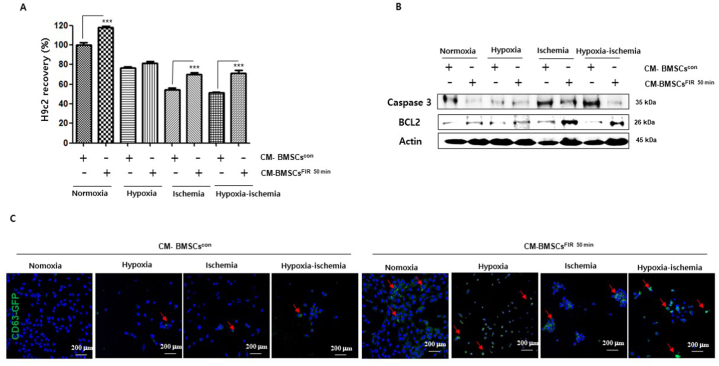
**The exosome derived from FIR-preconditioned BMSCs increases H9c2 survival against extra stress condition.** (A) After extreme environmental condition, graph of the damaged H9c2 cells were replaced with the CM- BMSCs^con^ and - BMSCs^FIR 50 Min^ for 72 h. The graph indicates the stress resilience of H9c2 cells. All data represent the mean ± SD of triplicated assays expressed as percentages of the BMSCs^con^. (B) Western blot analysis of caspase 3 and BCL2 expression in each group to access the apoptosis marker. Actin was used as a loading control. (C) After concentration of CD63-GFP-CM BMSCs^con^ and - BMSCs ^FIR 50 Min^, damaged H9c2 cells were incubated with CD63-GFP (green)-CM and - BMSCs ^FIR 50 Min^ for 72 h. Confocal images showed the CD63-GFP(Red arrow) and Hoechst33342-stained nuclei (blue). Scale bars, 200 μm. (For interpretation of the references to colour in this figure legend, the reader is referred to the Web version of this article.)

If FIR preconditioning boosts Akt-mTOR-MITF-exosome activation of cellular survival networks in BMSCs, preconditioned BMSCs could have more resilience than control BMSCs in the dynamic and stressful environments *in vitro*. BMSCs ^con^ and BMSC 50 min FIR were exposed to several extreme stress conditions: 4 °C, hypoxia, ischemia, and hypoxia-ischemia. After each condition, BMSCs ^con^ and BMSC 50 min FIR were incubated for 3 days, with the extent of their cell proliferation then measured using crystal violet staining, live cell staining with PI, and FACS analysis with Annexin V and PI. Under 4 °C, the cell viability of BMSC 50 min FIR was greater than BMSCs ^con^ (Fig. 5A and B). In the cases of hypoxia, ischemia, and both, BMSCs 50 min FIR were more resilient than BMSCs ^con^ (Fig. 5C and D). We extended our application of CM derived from BMSCs ^con^ and BMSC 50 min FIR to investigate the stress adaptation of H9c2 myoblasts, which is a cell line often used to test cardiac phenotypes in *in vitro* models [12]. H9c2 cells were subjected to hypoxia, ischemia, and hypoxia-ischemia conditions. H9c2 cells treated with CM-BMSCs 50 min FIR displayed more resilience than the control H9c2 cells, except under the hypoxia condition (Fig. 6A and B). However, FIR preconditioning did not appear to facilitate cell survival/proliferation under Western blot analysis further demonstrated the positive effect of CM-BMSC 50 min FIR on damaged H9c2 cells to detect caspase 3 and BCL2 expression (Fig. 6C). These results indicate that FIR preconditioning and CM improves the resilience and adaptation of BMSCs and H9c2 cells subjected to stress.

## 3 Discussion

The present study highlights cellular communications between MITF and mTOR-dependent signaling pathways, which are orchestrated responses to the signal amplification in FIR-preconditioned BMSCs. We found unexpectedly that the expression of MITF and exosome manufacture in BMSCs is regulated by FIR. Furthermore, FIR-preconditioning and CM-derived BMSCs 50 min FIR boost cellular resilience and adaption under extreme environmental conditions *in vitro*. In general, MITF is a member of the MiTF/TFE family of basic helix-loop-helix leucine zipper transcription factors. Recent papers suggest that the MiTF/TFE family serves an important role in organelle biogenesis, nutrient sensing, and energy metabolism [13]. Similar to all transcription factors, MITF governs multiple biological processes in the proliferation, cell survival, and differentiation of several types of cells with transcriptional collaborators [1,14]. MITF can target numerous post-translational modifications, including serine and tyrosine phosphorylation, ubiquitination, and SUMOylation [1,14]. Given the wide range of these biological processes, FIR-mediated phosphorylation of MITF at Ser 73 and of Akt at Ser 473 may be the starting point of signaling pathways to enhance BMSC function, including the subcellular localization, levels, and activity of MITF to activate transcriptional program.

Although the MITF-to-Akt-mTOR-exosome trajectory for enhancing stress resilience and adaptation in BMSCs had not been previously identified, several papers provide evidence for how changing the levels of MITF affects its functional collaborations with other transcriptional factors that lead to exosome generation and homeostasis in various cells [2]; d’Azzo A, 2020 [3]; Li W et al., 2021). For example, small molecule modulators such as fenoterol, N-methyldopamine, mephenesin, forskolin, and norepinephrine markedly increase exosome production in MSCs [2]. Small molecule modulators also significantly upregulate MITF expression [2]. Another paper demonstrated that MITF correlates with mTORC1 activity, RagD expression, and cell proliferation [15]. Our results found that FIR induced the expression of exosome via MITF and mTOR-mediated signaling pathways, which is agreement with previous observations [2]; Jones E, 2017). Using si-MITF, we show that the function of MITF is important in several seemingly uncorrelated cellular mechanisms that promote BMSC resilience such as in the Akt-mTOR-exosome manufacture system.

mTOR, a serine/threonine protein kinase, serves as a central hub of cell growth, proliferation, survival, and division in response to growth factors, nutrients, and the energy status of the cell [16,17]. mTOR can recruit other subunits to form two conserved signaling complexes; mTOR signaling complex 1 (mTORC1) and mTOR signaling complex 2 (mTORC2). In response to cellular energy homeostasis, amino acids, growth factors, and oxygen, mTORC1 regulates cell growth and metabolism through mRNA translation; synthesis of proteins, lipids and nucleotides; and catabolic processes such as autophagy [16,17]. mTORC2 governs cell survival and migration through phosphorylating glucocorticoid-regulated kinase, Akt/protein kinase B, and protein kinase C kinase families [[16], [17], [18]]. When activated, mTOR is phosphorylated on several residues such as Thr 2446, Ser 2448, and Ser 2481 [[16], [17], [18]]. For example, mTORC1 generally involves Ser 2448 in mTOR phosphorylation with binding partner raptor or rictor [[16], [17], [18]]. Ser 2481 is a sensitive marker for mTORC2 phosphorylation, as evidenced by being colocalized with the AMPK/mTOR/S6K1 signaling axis that resides in the mitotic and cytokinetic apparatus [[16], [17], [18]].

Our results indicate how FIR can contribute to preconditioned BMSCs by switching on relevant cellular signal pathways and transcription factors. CXCR_4_ and ERK pathways are involved in preconditioning BMSCs with FIR [9]. We observed that FIR triggered a phosphorylation cascade initiated by Akt ^Ser 473^, MITF ^Ser 73^, and mTOR ^Ser2481^ in preconditioned BMSCs. Eventually, the phosphorylated cascade of processes might lead to increases in BCL2 and HIF-1α, which promote BMSC survival. Although preconditioning signal leading to cellular protection through homeostasis is generally a well-known protective mechanism against neuroinflammatory damage, our findings might be involved in the relationship between the vitagene network and its possible biological relevance in the defense mechanisms against oxidative stress-driven degenerative diseases [[19], [20], [21]]. In numerous experimental models, natural antioxidants induce hormetic dose responses displaying endpoints of biomedical and clinical relevance. Interestingly, the mechanistic profile of natural antioxidants is similar to that of numerous other hormetic agents, indicating that activation of the Nrf2/ARE pathway is probably a central, integrative, and underlying mechanism of hormesis itself [[19], [20], [21]]. The Nrf2/ARE pathway provides an explanation for how large numbers of agents that both display hormetic dose responses and activate Nrf2 can function to limit age-related damage. This notion is consistent with experimental disease models, in which hormetic activation of Nrf2 effectively reduce the occurrence and severity of a wide range of human-related pathologies, including major neurodegenerative disorders [[19], [20], [21]]. Thus, the interplay and coordination of redox interactions with endogenous and exogenous antioxidant defense systems will be an emerging area of research interest in anti-inflammatory anti-degenerative therapeutics via the advantages of FIR-preconditioning.

### 3.1 Limitations of study

Our study has limitations that need to be considered. There is no direct evidence to decisively demonstrate the mechanism by which FIR-associated factors may regulate the MITF-Akt-mTOR-exosome trajectory in cell homeostasis of BMSCs. Further research focusing on FIR preconditioning and BMSC resilience could shed light on these mechanisms. Further research focusing on FIR preconditioning and BMSC resilience could shed light on these mechanisms. For instance, the combination therapy between FIR preconditioning and immune checkpoint inhibitor therapy in advanced melanomas might help to improve the therapeutic benefits between each efficacy and tolerability. It is well-known that MITF is an important determinant of melanoma cell plasticity and tumor heterogeneity which are undoubtedly one of the main hurdles for the powerful immunotherapy of melanoma [22]. Furthermore, a strategy for melanoma cells to gain a high proliferation rate is to avoid high MITF expression levels [22]. The merit of FIR-preconditioning might influence MITF-Akt-mTOR-exosome trajectory both in normal cells and the tumor microenvironment in melanoma. FIR-preconditioned normal cells might promote a cellular self-defense system to evade transformed cancer cells via MITF-Akt-mTOR-exosome trajectory. In summary, our findings identify how FIR preconditioning partially modulates a potential relationship between MITF and the Akt-mTOR-exosome manufacturing system in driving the resilience of BMSCs. These results indicate that FIR preconditioning has the potential to improve the effectiveness of BMSC therapies.

## 4 Materials and methods

### 4.1 FIR preconditioning and an array of environmental stress conditions

To establish the preconditioning of the BMSCs, we used a WS TM TY101 N emitter FIR therapy unit (WS Far Infrared Medical Technology CO, Ltd, Taipei, Taiwan) as described in Ref. [9]. BMSCs were exposed to FIR for 50 min (BMSCs^FIR 50 min^) to achieve sufficient preconditioning. Non-irradiated BMSCs (BMSCs^con^) were used as a control for all experiments. Environmental stress conditions applied in our study included low temperature, hypoxia, ischemia, and hypoxia-ischemia condition. H9C2, BMSCs ^con^ and BMSCs ^FIR 50 min^ were incubated under normoxic, hypoxia, ischemia, and hypoxia-ischemia conditions. Normoxic conditions consisted of 37 °C, 95% room air, and 5% CO_2_. Using a Galaxy 48 R incubator (Eppendorf, Hamburg, Germany), hypoxia was maintained at 37 °C, 95% N_2_ and 5% CO_2_ for 6 h with subsequent reoxygenation. Medium with no FBS or glucose was used to induce the ischemic condition as described in ^20^. Additionally, BMSCs^con^ and BMSCs^FIR 50 min^ were incubated at 4 °C for 1 h.

### 4.2 Cell culture and concentration of cultured medium

H9c2 cardiomyocytes were purchased from the American Type Culture Collection. Maintenance of BMSCs are described in Ref. [9]. The rat BMSCs of the P2 passages were used in all experiments. To concentrate the cultured media without FBS, 150 mm cell culture dishes were seeded with 1 × 10^7^ cells, preconditioned with/without FIR 50 min, incubated for 24 h, and concentrated from CM- BMSCs^con^ and - BMSCs^FIR 50 min^ using tangential flow filtration as previously described [7].

### 4.3 Transfection

BMSC-CD63-GFP cells were generated by transfecting BMSC cells with a pCT vector encoding for GFP fusion protein with human CD63 under CMV promoter (system Biosciences, CA, USA). siRNA-mediated down-regulation of MITF was achieved with a specific si-MITF (Fig. S3A). Scrambled siRNA was used as the control. Cells were seeded in 12 well plates for 24 h before transfection. Transfection was performed using Lipofectamin RNAiMax (siRNAs, Invitrogen) or Lipofectamin™ 2000 transfection reagent (Invitrogen; Thermo Fisher Scientific, MA, USA) according to the manufacturer's protocols. The cells were removed from the medium 24 h after transfection and placed in fresh medium containing 10% FBS and antibiotics. BMSC-CD63-GFP cells were characterized by confocal microscopy.

### 4.3 Cell proliferation and recovery assay

si-MITF BMSC and si-control BMSCs were exposed to FIR 50 min. After 3 days the cultured medium was removed, and then were stained with EZ-cytox solution for 1 h. The proliferation and recovery analysis of cell following plethora of environmental stress conditions was assessed using an EZ-Cytox cell viability assay kit (DoGEN, Seoul, Korea), crystal violet staining, and PI & Annexin V staining as described previously [9]. Absorbance was determined at 490 nm using an ELISA reader (Emax; Molecular Devices, Sunnyvale, CA, USA).

### 4.4 Quantitative reverse-transcription PCR (qRT-PCR)

cDNA was synthesized using AccuPower®RocketScript™ Cycle RT PreMix (dN12) (Bioneer, DaeJeon, Korea). qRT-PCR assays were carried out with SYBR®Green Mix and the appropriate primers (Applied Biosystems), and were run on a StepOnePlus real-time PCR system (Applied Biosystems). The relative gene expression from all data were obtained using the ΔCt method with normalization versus RPL-32 as previously described [[23], [25]]. The primers described supplementary information.

### 4.5 Immunofluorescence staining (IFS)

IFS was used to determine the expression of MITF, CD31, or mTOR after FIR 50 min preconditioning. The cells were fixed with 4% paraformaldehyde (PFA), stained with standard IFS methods as previous described [9,[23], [25]]. After nuclear Hoechst 33342 staining, immunostained confocal images were acquired using an inverted Zeiss Axio Observer Z1 microscope with 405, 458, 488, 514, 561, and 633 nm laser lines. Fluorescence labelling of exosome in BMSCs were labelled with the green-fluorescing, lipophilic dye PKH67 according to the manufacturer's recommendations (Sigma, St. Louis, MO, USA). To calculate the quantification of fluorescent-labeling of MITF, CD63, and CD63-GFP, we used microscope software ZEN from ZEISS microscopy which can count the number of fluorescent-labeling cells and number of total cells per field. All images were selected with sample identities blinded, and at least 20 random images were obtained from each well or group.

### 4.6 Western blot analysis

The samples were disrupted using the TissueLyser II (Qiagen), after which an ice-cold PRP-PREP protein extraction solution with a protease inhibitor cocktail (iNtRON Biotechnology, Inc, Seoul, Korea) was added, and the samples were homogenized by stainless steel beads (Qiagen, Cam USA). Protein concentration was assessed using the BCA-kit (Thermo Scientific, Rockford, IL, USA). An equal amount of protein (20 μg) from each sample was loaded onto 10% to 12% SDS gel, and transferred to a PVDF membrane (Merk Millipore, MA, USA). The membranes were blocked for 2 h at room temperature with 5% nonfat dry milk in PBS containing 0.1% Tween-20, and incubated with anti-MITF, anti-phosphor-MITF (S73), anti-mTOR, anti-phospho-mTOR (S2481) from Abcam (Cambridge, UK), anti-Akt, anti-phospho-Akt (S473), anti-phosphor-AMPK (Thr172), anti-FOXO3α, anti-phosphor-FOXO3α, BCL2, actin, and HIF-1α from Cell Signaling technology (Danvers, MA, USA) (1:1000) overnight at 4 °C. After washing three times, the membranes were incubated with a horseradish peroxidase-conjugated secondary antibody (1:5000) at RT for 2 h and visualized with a chemiluminescence substrate.

### 4.7 Statistical analysis

Student's *t-*tests (for comparisons of two groups) or a one-way analysis of variance (ANOVA) (for comparisons of three or more groups) followed by Tukey post hoc tests were used for the statistical analyses. SPSS software ver. 17.0 (SPSS, Chicago, IL) was used. A value of *P<0.05* was considered significant. Data are expressed as means ± standard error of the mean (SEM). Data analysis was carried out using the GraphPad Prism software (GraphPad Software Inc). *P<0.05 – 0.01, **P<0.01 – 0.001, and ***P<0.001 vs. corresponding controls. All error bars represent the standard deviation of three or more biological replicates.

## Author contributions

J.Y.M and K.W. contributed to the conception or design of the research. J.Y.M performed the experiments; analyzed the data and wrote the paper. J.Y.M and K.W edited the manuscript, and we designed the research. J.Y.M and K.W. are the corresponding authors. All authors read and approved the final manuscript.

## Funding statement

This research was funded by Priority Research Centers Program through the National Research Foundation of Korea (NRF) funded by the Ministry of Education, Science and Technology (2022R1A2C2008867, 2017R1A6A1A03015562, and 2020R1I1A1A01054595).

## Declaration of interests

All authors declare no competing interest.
