# Supplementary material for: FIR-preconditioning promotes Akt-mTOR-exosome manufacture in cooperation with MITF to boost resilience of rat bone marrow-derived stem cells
Source: Heliyon. 2023 Apr 7;9(4):e15003. doi: 10.1016/j.heliyon.2023.e15003 (PMC10130773; doi:10.1016/j.heliyon.2023.e15003)

**FIR-preconditioning promotes Akt-mTOR-exosome manufacture in cooperation with MITF to boost resilience of rat bone marrow-derived stem cells.**

Yun-Mi Jeong^1^, Weon Kim^2*^

^1^Department of Mechanical Engineering, Touch University of Korea, 237 Sangidaehak Street, Si-heung City, 15115, Republic of Korea

^2^Division of Cardiology, Department of Internal Medicine, Kyung Hee University Hospital, Kyung Hee University, Seoul, Republic of Korea

*co-corresponding authors contributed equally to this work. Correspondence: [phdjeongym12@tukorea.ac.kr](mailto:phdjeongym12@tukorea.ac.kr)(Y.-M.J.); mylovekw@hanmail.net(W.K)

**Materials and methods**

**Cell viability**

To assess si-Con and si-MITF BMSC viability, the crystal violet staining assay was used. Briefly, After transfection for 24 h, the culture medium was removed. Si-Con and si-MITF BMSCs were stained with 0.1% crystal violet solution (Sgma, St Louis, MO, USA) room temperature, then washed four times. The crystal violet retained by the adherent cells was extracted with 95% ethanol. The absorbance was determined at 590 nm using an ELISA reader(VERSAMax; Molecular Devices, Sunnyvale, CA, USA).

**Reverse-transcription PCR (RT-PCR)**

cDNA was synthesized using AccuPower®RocketScript^TM^ Cycle RT PreMix (dN12) (Bioneer, DaeJeon, Korea). RT-PCR assays were carried out with PCR thermal cyclers and the appropriate primers (Applied Biosystems). RPL-32 housekeeping gene is used as internal control for mRNA expression. The primers described as below;

|  | Forward | Reverse |
| --- | --- | --- |
| RPL32 | TGTCAAGGAGCTGGAAGTGC | AGGCACACAAGCCATCTATTCA |
| mTOR | TCTTCCAGCAAGTTCAGCCC | GAATCAGACAGGCACGAAGG |
| MITF | CATCACGCATCTTGCTACGC | TGCATGAACTGGGCTGCCTG |
| BCL2 | ATCGCTCTGTGGATGACTGAGTAC | AGAGACAGCCAGGAGAAATCAAAC |
| HIF1α | GCCCCAGATTCAAGATCAGCC | CGCTGTCCACATCAAAGCAGT |

**Nonstandard abbreviations**

**BMSC**

bone marrow-derived stem cell

**FIR**

far-infrared irradiation

**MiT/TFE**

microphthalmia family of bHLH-LZ transcription factors

**MITF**

microphthalmia-associated transcription factor

**CM**

conditioned medium

**BMSCs^FIR 50 min^**

BMSCs were exposed to FIR for 50 min

**BMSCs^con^**

non-irradiated BMSCs

**IFS**

immunofluorescence staining

**Figure S1. FIR preconditioning affects the expression of MITF and mTOR-BCL2-HIF1α at mRNA levels.** After FIR ^50 min^ preconditioning, total RNA was prepared at the indicated time point. RT-PCR to quantify the expression of the targeted genes in BMSCs^con^ and BMSCs^FIR 50 min^.


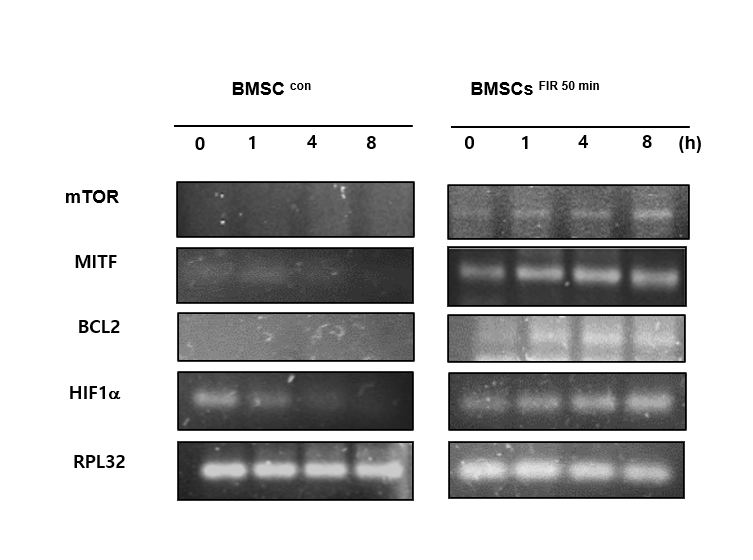


**Figure S2.** **Exosome mRNA in BMSCs is elevated by FIR preconditioning.** (A) After FIR ^50 min^ preconditioning, total RNA was prepared at the indicated time point. RT-PCR to quantify the expression of the CD63 in BMSCs^con^ and BMSCs^FIR 50 min^.


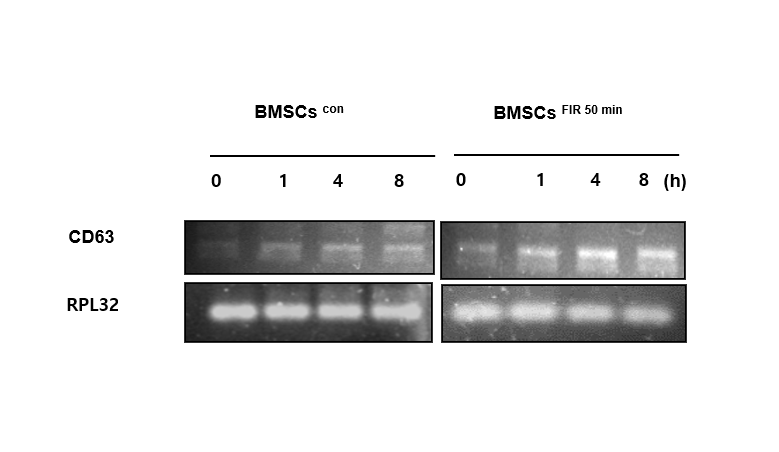


**Figure S3. Down-regulation of MITF with or without si-MITF.** (A) qRT-PCR to quantify the expression of the MITF in si-con BMSCs and si-MITF BMSCs.(B) The cell viability of si-con BMSCs and si-MITF BMSCs using the crystal violet staining method.


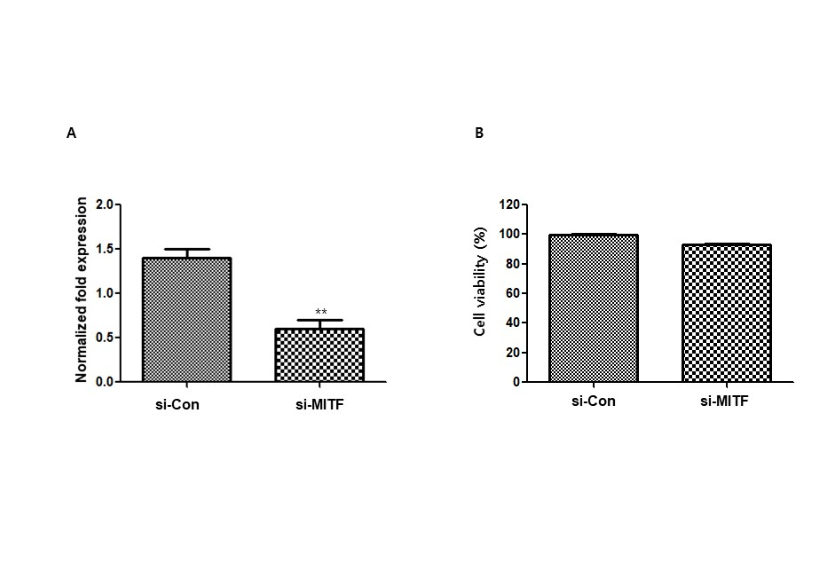


**Figure** **S4. Down-regulation of mTOR, MITF, and exosome by FIR with or without si-MITF and rapamycine.** Western blot analysis of mTOR, CD63, BCL2, and MITF in si-Con BMSCs^con^, si-Con BMSCs^FIR 50 Min^, si-MITF BMSCs^con^, and si-MITF BMSCs^50 Min^ with or without RA(rapamycine).


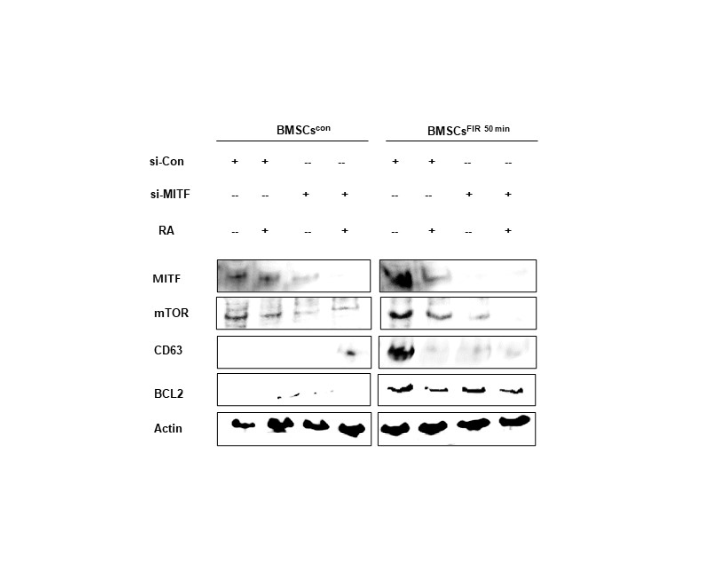


The supplementary part including western data(Fig 1B, 2B,3B, 6B in the manuscript.


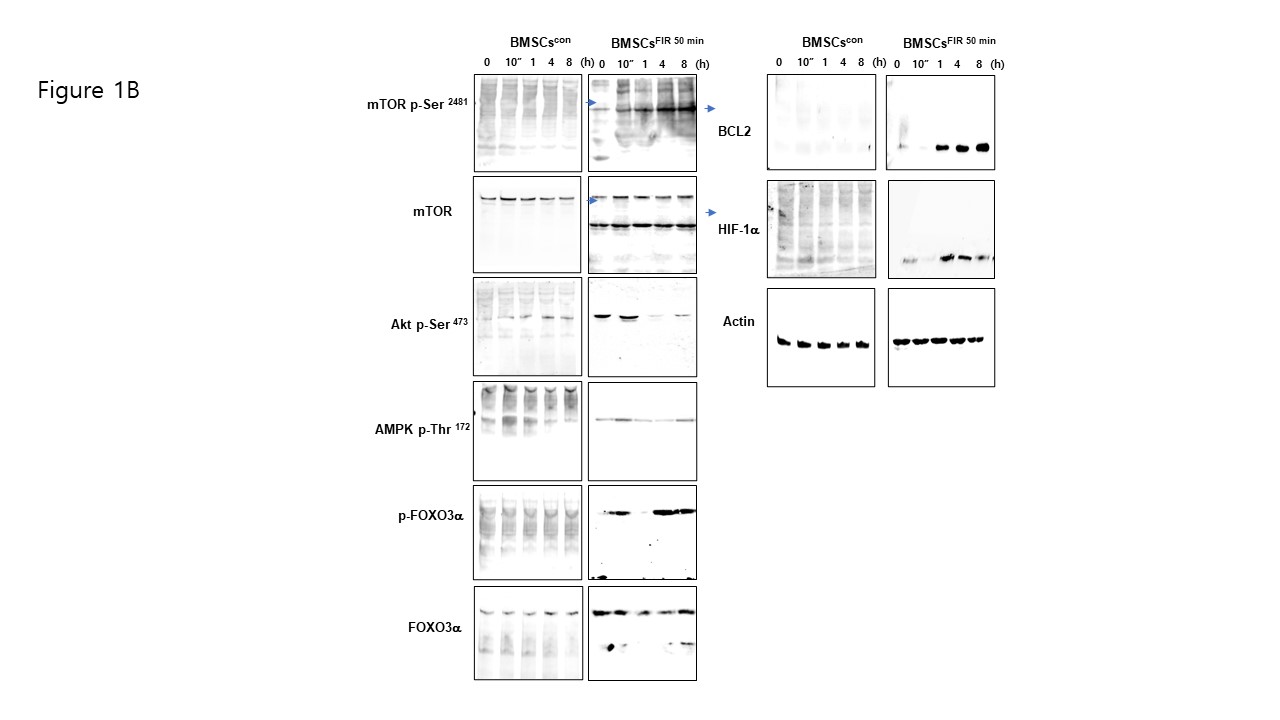

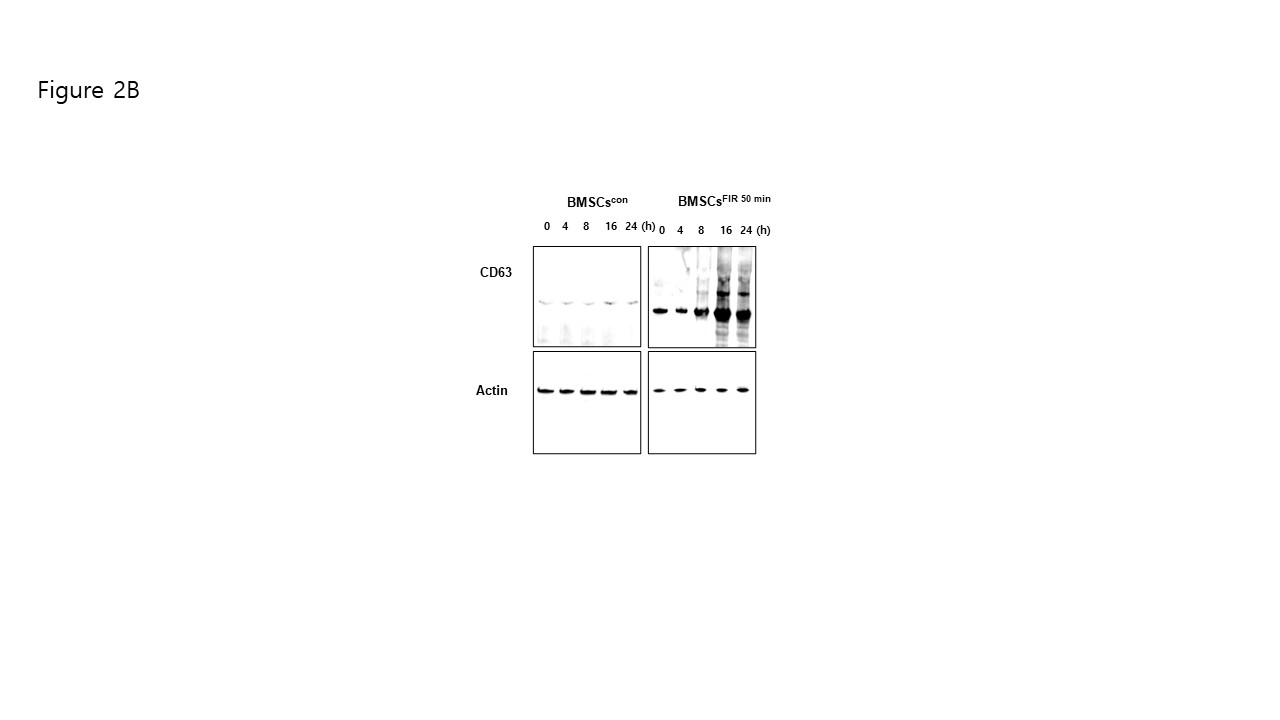

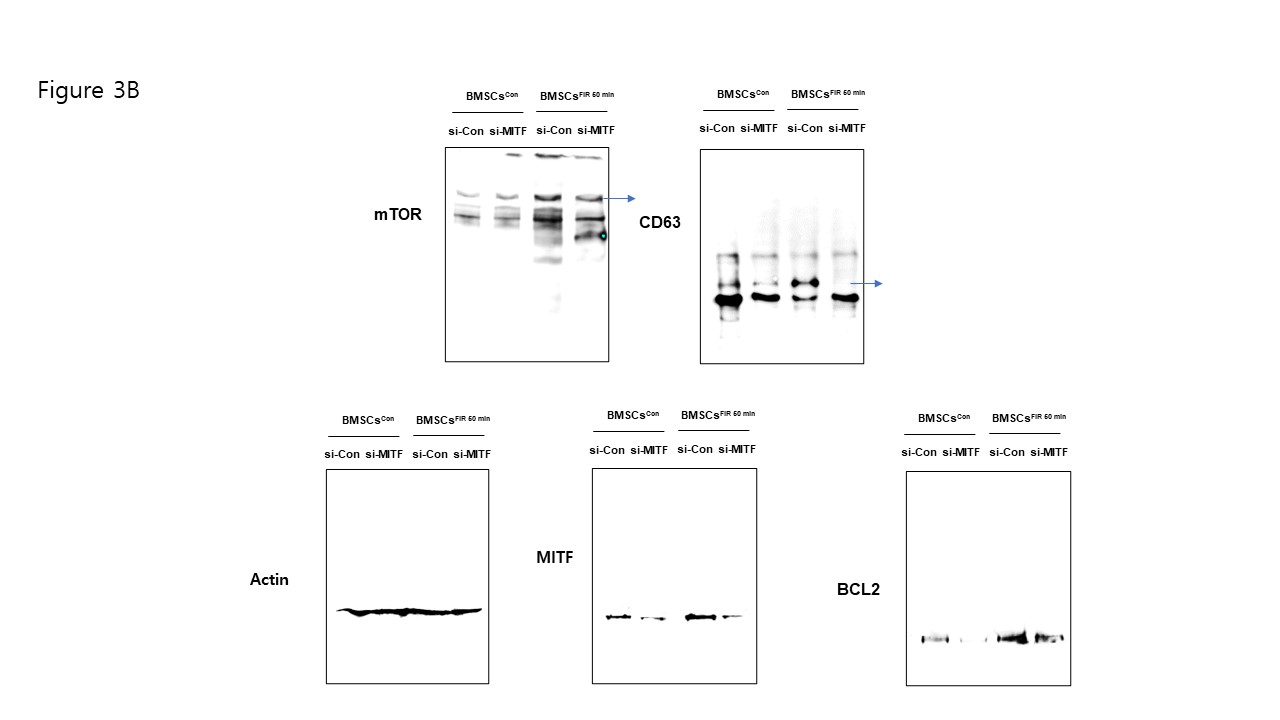

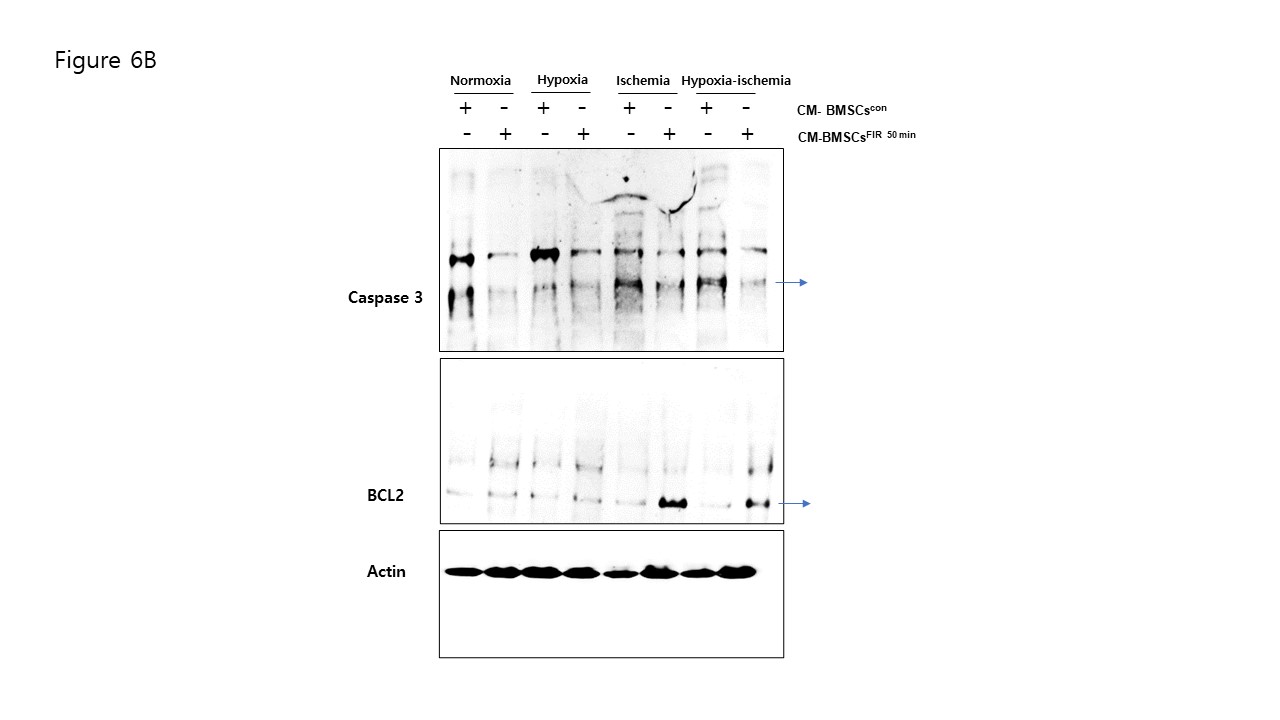

Supplement: Multimedia component 1 [file mmc1.docx]
